# Challenges and opportunities for perinatal health services in the COVID-19 pandemic: a qualitative study with perinatal healthcare professionals

**DOI:** 10.1186/s12913-022-08427-y

**Published:** 2022-08-12

**Authors:** Bettina Moltrecht, Simone de Cassan, Elizabeth Rapa, Jeffrey R. Hanna, Clare Law, Louise J. Dalton

**Affiliations:** 1 Department of Psychiatry, Warneford Hospital, University of Oxford, Oxford, OX3 7JX UK; 2 Evidence-Base Practice Unit, University College London, London, N1 9JH UK; 3 Centre for Longitudinal Studies, University College London, London, WC1H 0NU UK; 4 School of Nursing and Midwifery, Queen’s University Belfast, Belfast, BT9 7BL UK; 5 Centre for Early Child Development, Blackpool Better Start (NSPCC), Blackpool, UK

**Keywords:** Young parents, COVID-19, Perinatal, Healthcare professionals

## Abstract

**Background:**

Perinatal healthcare professionals (PHCPs) provide essential support to all parents in the perinatal period, including young parents aged 16–24, who are at an increased risk of morbidity and mortality. Little is known about the impact of COVID-19 restrictions on the provision of perinatal services, and on perinatal healthcare professionals, caring for young parents in the UK.

**Methods:**

A UK based qualitative study using semi-structured interviews with perinatal healthcare professionals (*n* = 17). Data were analysed using thematic analysis.

**Results:**

Two themes were identified describing perinatal healthcare professionals’ perceptions of providing care to young parents during the pandemic. Perinatal healthcare professionals perceived that young parents’ needs were amplified by the pandemic and that pandemic-related changes to the service, such as the use of telemedicine to replace face-to-face interactions, did not manage to successfully mitigate the increased feelings of anxiety and isolation experienced by young parents. Concerns were raised by perinatal healthcare professionals that these changes reduced young parent’s access to vital support for themselves and their child and may contribute to exacerbating pre-existing inequalities.

**Conclusions:**

This study provides insight into the impact of the COVID-19 pandemic on the provision of perinatal care to young parents. Perinatal mental health professionals felt these negative impacts could be overcome by using a blended approach of technology and face-to-face interactions allowing regular contact with young parents and facilitating the exchange of vital information, while maintaining access to opportunities for social interactions with other parents. Findings from this study could be used to future-proof services against further COVID-19 restrictions.

## Introduction

The COVID-19 pandemic has led to substantial changes in perinatal care provision in the United Kingdom (UK) and across the world [1–3]. Perinatal care services play a vital role in supporting parents, especially mothers, during pregnancy and their first postpartum year [4]. In the UK perinatal services are essential in identifying parents’ and infant’s needs, making onwards referrals to relevant services, and providing ongoing support via Health Visitors and the Family Nurse Partnership. Young parents (aged 16–24) are provided with enhanced support by these services as it has long been recognised that this population is at risk of increased morbidity and mortality, both to the mother as well as the infant [5–7].

There have been concerns about the impact of the COVID-19 pandemic on parents during the perinatal period [3, 8, 9]. A growing body of global evidence has highlighted the greater levels of psychological distress, anxiety, and depression in perinatal populations during this time [10–13]. Mothers and fathers have been extremely worried about the potential consequences of COVID-19 infection on the mother, the foetus or infant [9, 14]. Furthermore, COVID-19 restrictions have led to reduced contact and support from families, friends and health and social care services [8, 14]. Evidence from a recent UK wide survey has suggested that young parents and those with little or no income have been at even greater risks of experiencing mental health difficulties during the pandemic than older parents or parents with a higher income [10, 11, 15, 16]. This is of particular concern as poor parental mental health has repeatedly been shown to adversely affect parent and child outcomes [17].

In addition to the increased risk posed to families by the COVID-19 pandemic, increasing evidence suggests that healthcare professionals themselves have experienced significant challenges during this time while continuing to provide support to this vulnerable population. This includes reports of increased anxiety relating to occupational exposure to COVID-19, increased workforce pressures, as well as changes to the provision of services themselves such as a transition to remote working and reduced contact with patients [2, 18–21]. For example, a large survey of perinatal mental healthcare professionals identified their concerns about the impact of pandemic-related remote working on their ability to robustly assess maternal-infant relationships or domestic violence [3]. A recent global survey with 714 maternal health workers indicated that 90% reported ‘somewhat’ or ‘substantially higher’ stress levels [22]. Furthermore, most survey participants reported a reduction in maternal care services and significant changes to care practices, which some felt were not evidence-based.

Providing safe and high-quality perinatal care is important to both the parents and perinatal healthcare professionals (PHCPs) involved in their care. However, most studies investigating changes during the COVID-19 pandemic so far have focused on hospital and acute care settings, have been predominantly based outside of the UK and used a survey-based approach [18, 20–23]. It remains unclear how PHCPs in the UK and across hospital and community settings have experienced COVID-19 pandemic-related challenges. Investigating PHCPs’ experiences of delivering care during the pandemic provides insights into their perception of how changes in service provision impacted on both them and vulnerable groups across the country.

### Aims and objectives

This study explores PHCPs’ experiences of providing care to young parents during the COVID-19 pandemic in the UK. We aim to investigate:

1. PHCPs' perceptions of the challenges in providing care to young parents during the COVID-19 pandemic.
2. How PHCPs navigated the increased challenges in providing care during the COVID-19 pandemic.

## Methods

This is an exploratory qualitative study using semi-structured in-depth interviews. The study is reported following the Standards for Reporting Qualitative Research framework [24].

### Participants

Using convenience and volunteer sampling techniques, seventeen PHCPs were recruited between November 2020 and May 2021. Participants were eligible if they have been working with young parents (age between 16 and 24 years) during the COVID-19 pandemic. These professionals were involved in perinatal care services in the UK during the COVID-19 pandemic. Although the research team asked specifically about their experiences of working with young parents, some professionals highlighted that they were working with parents of all ages and that it was sometimes difficult to report distinctly on their experiences with young parents.

### Study recruitment

Participants were invited to take part in a one-off interview by responding to an advert. Study adverts were developed by the research team and a) distributed via email to existing contacts of the research team and b) shared via Facebook and Twitter by team members and through relevant professional networks and organisations that have an interest in or provide parental mental health support in the UK, such as perinatal mental health teams, health visitor services, midwifery networks, and community groups.

The study advert included a link to access an online participant information sheet, providing individuals with more details on why the study was being conducted, what their involvement would be and the risk involved to taking part. Willing individuals then completed an online form to express their interest in taking part in the study. Interested individuals were contacted by one of the authors via email, to assess if they met the eligibility criteria and agree a date and time for the interview. Individuals provided informed written consent prior to the interview.

### Data collection

Interviews were conducted by two researchers [BM, LJD], with the majority being conducted by the first author [BM]. A semi-structured interview guide (see Table 1) was developed informed by the aims and objectives of the study as well as the prior experience of the research team. The interviewer produced detailed reflections after each interview and were discussed with four co-authors throughout the data collection period [BM, JRH, LJD, ER]. Following reflexive team discussions, the guide was iteratively reviewed throughout the study. All interviews were conducted via Microsoft Teams, audio recorded, and later transcribed by an external provider. Interviews lasted between 12 and 82 min (mean average = 47.1 min). Data collection was terminated once no further categories were identified by the authors involved during the data collection period [BM, JRH, LD, ER].

**Table 1:** Semi structured topic guide for interviews

Changes to working practices during COVID-19 pandemic
• Challenges and positives arising from pandemic
Changes observed for young parents during COVID-19 pandemic
• Impact on mothers and on fathers
• Impact of pandemic on parental mental health
Strategies employed to adapt services and support parents during pandemic

### Data analysis

Audio-recordings were transcribed verbatim and managed using NVivo V.12. Data were analysed using an inductive coding approach following the principles of reflexive thematic analysis by SdC and BM [25, 26]. Initially BM and SdC read and re-read all of the transcripts while taking notes. Coding of the first five transcripts created an initial list of codes which were discussed and refined with the team through critical dialogue. Themes were identified based on the list of codes and organised into themes with the help of mind-mapping activities [25, 26] and discussion with the research team.

### Reflexivity, research group and context

The research team are all of white ethnicity. SdC identifies as female and is a Psychiatry Trainee working within the National Health Service (NHS) who has experience of working with adults and children in a mental health setting. BM identifies as female and is a Clinical Psychotherapist who trained outside of the UK and has experience working with children, parents and families across various clinical and research settings and currently works as a post-doctoral researcher in the UK. ER identifies as female and is a post-doctoral researcher, who has conducted extensive research on parental mental health and childhood outcomes. JRH identifies as male and is a Masters Nursing student and researcher with expertise in qualitative research. CL identifies as female and is a senior leader in services delivering support to deprived communities. LJD identifies as female and is a Consultant Clinical Psychologist who has worked extensively with parents in the perinatal stage.

Four authors have experience of delivering clinical services to adults and children (BM, SdC, JRH and LJD) within an NHS context (SdC, JRH and LJD).

Data collection took place from November 2020 to May 2021. In the UK, the first lockdown was announced on 23^rd^ March 2020 and lasted until the beginning of June 2020. A second and third lockdown followed and lasted from November 2020 until March 2021.

### Ethical considerations

Participants were given the option to complete the interview on the telephone or via the online platform Microsoft Teams. Participants provided written and oral consent prior to taking part. No participant withdrew from the study and no significant distress was reported in response to the interview process. Individuals were reminded that they could withdraw at any time without giving a reason. All potentially identifying information about participants was removed from transcripts to protect participants’ anonymity. The study received ethical approval from the Research Ethics Committees of the University of Oxford (R72496) and the National Society for the Prevention of Cruelty to Children (R.20.193).

## Results

### Participants

A total of seventeen PHCPs from across the UK were included in this study. Participants were all female and had experience of working with parents prior to and during the COVID-19 pandemic. A range of professionals were included in this study and are outlined in Table 2.

**Table 2:** Roles of perinatal health care professional participants

Professional Role	Number
Health Visitor	4
Family Nurse Partnership Healthcare Professional	5
Perinatal Mental Health Professional	2
Midwife with specific responsibility for teenage parents	1
Midwife	1
Social Worker	1
Specialist Nurse Sonographer	1
Specialist Nurse for Newborn and Infant Examination	1
Youth Worker running community courses and support programmes	1

PHCPs described how pandemic-related changes to perinatal services affected their ability to provide the same level of care to parents as before the pandemic. In particular, PHCPs reflected how young parents faced pre-existing challenges*,* when compared to older parents, which increased their vulnerability to the pandemic-related systems changes experienced by services. Interestingly, despite these observations professionals did identify positive changes to perinatal services as a result of the COVID-19 pandemic. Overall, two themes were identified and further categorised into four broad subthemes as shown in Table 3.

**Table 3:** Overview of identified themes

Themes	Subthemes
1. Perinatal healthcare professionals’ perceptions of how young parents' needs were amplified during the pandemic	A) Working with the challenges faced by young parents B) Specific pandemic-related challenges for young parents
2. Perceptions of the impact of COVID-19 on delivery of perinatal care	A) Delivery of perinatal care using virtual and remote methods B)Consequences of changes to perinatal care

## Theme 1: Perinatal healthcare professionals’ perceptions of how young parents' needs were amplified during the pandemic

PHCPs often reflected on the challenges faced by young parents, even before the advent of the pandemic, commenting on their contrasting experiences of providing care for younger and older parents. Some of the areas highlighted by PHCPs related to younger parents’ socioeconomic status and perceived role of healthcare professionals providing support and guidance to young parents. These issues are further discussed in the two identified sub-themes.

### Subtheme A) Working with the challenges faced by young parents

Participants believed that young parents had often been exposed to more adverse childhood experiences (ACEs). This included the presence of complex family relationships, being subject to involvement of other statutory agencies from a young age, such as social services, as well as having a high rate of pre-existing mental health difficulties. These factors were perceived by PHCPs to differentiate this cohort from older parents and to make younger parents a more ‘*vulnerable group*’.

‘I mean we work with young first-time teenaged parents so they are a vulnerable group in any case. A lot of that is based on family issues in the past, a lot of them have got multiple ACEs so there’s been a historical childhood impact on them from family.’ (P008, Family Nurse)

Those professionals who are involved in home visits as part of their role described some young mothers’ home environments as being *‘overcrowded’* and being comprised of ‘*a few generations all living together’*. Professionals reflected that this created challenges for key aspects of their practice; for example, it was often difficult to identify suitable spaces for clinical interactions that enabled confidentiality to be maintained. Some professionals reflected this may have prevented young parents from feeling able to *‘talk about difficulties that they are experiencing’*. While some professionals identified this as an issue pre-pandemic, it was felt that the pandemic and the subsequent lockdown exacerbated this problem.

“A lot of our younger women are living with their family, so actually to try and find that space by themselves is, is really hard to do, and some of those younger women struggled a bit with those relationships with their family as well. So, actually, (A) it’s trying to find that space, but then, (B) there is that, always that worry that you’ll be overheard” (P004, Perinatal Mental Health Professional)

PHCPs reflected on the relationships present in the lives of young mothers. Some PHCPs reported that young mothers were often no longer in a relationship with the father of their child, or they were not living together. Consequently, young parents often faced parenthood on their own or relied on other family members to provide them support in the perinatal period. Some participants also reflected that the contrast between the day-to-day lives of some young parents and their peers who were not parents left them feeling alone and isolated. PHCPs felt this situation was exacerbated by the COVID-19 pandemic and may have contributed to younger parents creating closer ties with healthcare professionals.

“…..if you're the first in your peer group who has become pregnant at the age of 18 or 19. It’s not an experience that your peers, the people that you met to school, university, college with and you’ve grown up with actually have a lived experience of… certainly, in the community there’s a much stronger bridge between the healthcare professionals and new mums coming through.” (P011, Midwife)

### Subtheme B) Specific pandemic-related challenges for young parents

PHCPs felt some new or expectant young mothers were able to successfully navigate the perinatal period. However, more often professionals felt that pregnancy and the post-partum time were a particularly challenging period for younger mothers. For example, some professionals felt young mothers did not know how to “*access information out there*”. PHCPs felt young mothers often had less ‘*confidence*’ to ask professionals for this information and would therefore ‘*ask for less’* when contrasted to older mothers.

“..for some of our younger ladies it felt like, because they’ve not had that level of, of life experience, it was harder for them to ask for what they wanted and they were happy to be guided by, by the team more, I suppose. They were less, they were less likely to say, “This is what I want, this is what I want,” and they were happier to be led” (P004, Perinatal Mental Health Professional)

This worried some professionals who felt that young parents, especially first-time parents, might be unaware of their own or their child’s health needs. Participants expressed concern that changes to perinatal service delivery during the pandemic may have exacerbated this issue.

“They feel they can’t ask for support because they don’t know that they need support” (P015, Health Visitor)

Professionals felt that the care they were providing to young parents, particularly when they were able to see some patients face-to-face again, was essential in terms of supporting mothers because ‘*they weren’t necessarily getting that support from other place(s)’*. More broadly, PHCPs felt a lack of psychosocial support may even exacerbate social inequalities as many ‘*free services’* run by local councils were not available. It was felt that some parents *‘worked their whole life around things that were being offered’* by services, whereas ‘*older mums*’ would be able to pay to access private group and services.

“I think a loss of things like the children’s centres and mum and baby groups …..the more, sort of, voluntary sector, third sector agencies that, that used to do a lot of that valuable work. That’s been really challenging so they’ve not had the drop-in groups, children’s centres to go to, mum and baby things” (P002, Perinatal Mental Health Professional)

Most professionals noted that younger parents placed a greater emphasis on receiving consistency of care relative to their older counterparts. Consequently, several expressed concerns about how reductions to the level of face-to-face care resulting from the pandemic would impact young parents. Some young parents who were identified as having other pre-existing co-morbidities, such as mental health difficulties or complex family dynamics, wanted to see the same professional at each contact and found speaking to different team members challenging. Professionals voiced different potential reasons driving this such as young parents being *‘frustrated’* at having to repeatedly retell their story to different members of a team, or keeping things more superficial as *‘they* [young parents] *struggle with communication and expressing how they feel’*.

“Because if you haven’t got that [trusting relationship], you are not going to get any depth with them much at all. But if you’ve got that and they trust you and they’ve got, if you’ve got that unconditional professional love for them and a professional love but you know what I mean, you’re there whatever. A lot of them haven’t had that and they, yes they will go a long way with you if you can build that up.” (P005, Family Nurse)

## Theme 2: Perceptions of the impact of COVID-19 on delivery of perinatal care

Professionals reported an increase in their workload due to the re-deployment of professionals to different services as well as colleagues having to self-isolate during the COVID-19 pandemic. Other challenges identified included adapting to rapidly changing infection control policies, the use of personal protective equipment (PPE), and fears for their own safety at work in terms of exposure to COVID-19*.* PHCPs reported that the shift to remote working and the impact of social distancing guidelines had affected the provision of perinatal care, particularly for young parents. These are discussed in the two sub themes below.

### Subtheme A) Delivery of perinatal care using virtual and remote methods

Professionals discussed how switching from face-to-face working to virtual and remote methods meant women had to attend hospital appointments alone and that many community services, (such as mother and baby groups and clinics) were shut. Navigating this change was further complicated by rapidly changing guidelines, the need for policies to be updated before the adapted care could be delivered, as well as limited resources within services to provide PHCPs with suitable devices, such as smart phones. Most PHCPs stated that they perceived this system change as ‘*a big disruption’* to providing care to their clients and left some professionals with *‘a feeling of frustration’.* As a result, PHCPs had to adapt how to deliver care to their patients by re-designing material or finding other ways of supporting parents remotely. On reflection, this was described as *‘not a smooth transition’* by PHCPs as certain elements of care could not be replicated online.

“I have a big kit that I carry around in a pillowcase full of tools to demonstrate labour and birth and trying to do that over a camera, yes it’s difficult.” (P006, Teenage Midwife)

Many PHCPs felt that particularly in the early phases of the pandemic service provision moved from seeing parents in person or providing support groups and targeted interventions to sign-posting mothers to information or delivering generic care packages. Despite the fact that many services continued to function to some level during the pandemic, it was felt that the changes altered the care that was provided to parents from preventative work to focusing on responding to crises.

“(the service)..it would have been more proactive before. It would have been- Because these mothers would have been coming to the lounge, playgroups, baby massage. They- they would have more visits from us. And we would’ve seen things earlier, you know” (P014, Health Visitor)

While PHCPs perceived that some parents tried to embrace new technologies in order to continue accessing services, many PHCPs felt parents were also more likely to ‘*avoid’* or ‘*cancel’* remote appointments. Additionally, young parents were often reported to not have the technology or financial resources to access remote perinatal services. Professionals also described their surprise that despite thinking younger parents would be ‘*tech savvy’* some appeared to struggle more in terms of accessing and engaging with online appointments. Some PHCPs linked this back to ‘*a lack of confidence*’ in this cohort, leaving PHCPs with specific worries about young parents accessing support.

“Some [young parents] prefer the digital world and will text you and talk to you on the telephone rather than face to face, others are very anxious about telephone contact…” (P007, Family Nurse)

Virtual interactions were often perceived unsuitable for consultations, particularly where parents did not have sufficient technology or financial resources for internet access; this was observed to be particularly prominent among young parents.

There were professionals that expressed concerns that parents felt uncomfortable sharing their difficulties with professionals over the phone/online; this was attributed to the challenge of building rapport and establishing a trusting relationship that was felt to be particularly important for young parents accessing care.

On occasion, some professionals felt that young parents delayed much-needed assessments or interventions until they could be delivered face-to-face rather than online interactions.

‘She says, “I can’t do it anymore on the phone.” I say, “Look just wait for me next week”’ (P010, Family Nurse)

Many PHCPs felt their assessments were ‘s*uperficial*’ when conducted over the phone and provided fewer opportunities for the professional to identify concerns related to how the mother was coping, the child itself and the home environment. This led to concerns from some PHCPs about whether they had ‘*missed anybody’* in terms of identifying children’s needs. This was a particular worry for children of young parents who were often navigating this period with less support, awareness of their needs or confidence to ask for help (reported in Theme 1 sub theme B).

“…..especially with the younger mums it’s around what you learn from just laying eyes on them, … having that face-to-face interaction, how they communicate, how they interact……. you learn a lot by the fact of can they get to clinic, bring a child that’s looking reasonably clean and tidy, manage the appointment to get there roughly on time, leave again.” (P002, Perinatal Mental Health Professional)

Although it was felt by PHCPs that technology decreased some parents’ engagement with services, for others it resulted in improved outcomes as they were ‘*getting them on the phone or getting them on the internet anywhere’.* Some participants also reported finding WhatAapp a new and useful format to communicate with parents. However, participants reflected that online contact appeared to work best when professionals had already ‘*established a relationship with a face to face [visit] in the house’*, which was then continued online. Such a ‘*blended approach’* was felt to increase choice for patients which ultimately could enhance their care. However, participants also emphasised the need to consider how to successfully build relationships with parents before moving online.

“we were in constant touch with our mobiles saying we can’t come and visit this week but how would you like to do this, would you like a phone call, would you like an Attend Anywhere so we had maintained that contact all the way through. Like I say we’ve had more contact in actual fact” (P008, Family Nurse)

Although an increase in workload was described, many professionals welcomed the pandemic ‘*pushing’* them to make adaptations to delivering care online. Less time travelling between homes meant some PHCPs felt they had more time to interact with parents more regularly, particularly if they were able to utilise a blended approach of face-to-face and technology-based contact.

“So there’s actually more time to make that phone call say for instance with a client. You know 10 min just checking in, seeing how you are.” (P008, Family Nurse)

PHCPs described that they tried to be more ‘*creative’* in the way they were using technology for instance by engaging parents through private Facebook groups, and enabling parents to connect with PHCPs more regularly or to highlight any immediate concerns.

“So I absolutely love my mobile phone for work. I have mums send me videos on it…. I have a look at it, I give them a lot of help and support around what they need to look for……I think my mums feel very reassured by the fact they can just text me” (P015, Health Visitor)

PHCPs identified the benefits of technology coupled with pandemic restrictions resulting in fathers being more likely to be at home, which allowed PHCPs to include them more in appointments, as many voiced fathers were a ‘*massive area that’s overlooked*’. However, participants felt this was more common in mothers who were older and had a more stable family unit, rather than younger mothers who might not be in a relationship or living with the child’s father.

“So, it was nice in a way that they were home to include them more in the visit and often you know they would be, on the Attend Anywhere, they would be sitting by the… partner and participating and getting the information as well or joining in or saying you know, “It’s tough” or, “It’s great” or whatever.” (P012, Health Visitor)

Where technology was not appropriate, some Health Visitors described instigating walks with young parents as an alternative to virtual contact. Walks were reported as helpful as they allowed PHCPs to follow social distancing guidelines while maintaining in person contact with the young parent. While this intervention was described as helpful to mothers, some reflected it required *‘careful negotiation’* for those who may not routinely engage in this type of activity*.*

“When you are just walking side by side with somebody you can have conversations that don’t feel as challenging or as intense as if you were just sat in the room face to face, I think” (P008, Family Nurse)

### Subtheme B) Consequences of changes to perinatal care

PHCPs felt that government social distancing guidelines meant there was a lack of opportunity for young mothers to *‘bond’* with other parents, leading to increasing feelings of social isolation during the pandemic.

A lack of social interaction was considered by professionals to be linked to an increase in mental health problems across the board resulting in mothers becoming more ‘*anxious’* and ‘*depressed’;* this was described as prominent with young parents who were also experiencing pre-existing difficulties. PHCPs felt that at times this led to parents engaging more frequently with perinatal services to meet their needs.

“all the nurses are coming back to me and saying the contacts have actually increased. So although they’ve not done as many face to face, they’ve done more telephone or virtual contacts to try and alleviate some of those anxieties. Because sometimes in a lot of those cases we are the only professional that’s working with them.” (P008, Family Nurse)

It was often felt that prior to the pandemic, face to face sessions had offered parents important opportunities for socialising with a peer group; participants felt that online interactions did not provide an adequate substitute.

“So then having a group online and some of our ladies were saying the issue was, it’s not the same as being able to just have these little conversations with people.…our younger ladies as well just said that they felt very uncomfortable using online, and that it just wasn’t something that they found helpful.” (P004, Perinatal Mental Health Professional)

## Discussion

Perinatal healthcare professionals described several challenges providing care to young parents during the COVID-19 pandemic resulting from the introduction of UK governmental social distancing guidelines which led to a reduction of face-to-face contact as well as the closure of community perinatal as well as third sector services [27]. PHCPs described young parents as being particularly impacted by these service changes as they represent a distinct, and often more vulnerable patient cohort under their care. This is consistent with extensive research showing young parents face higher levels of socio-economic deprivation, have higher rates of perinatal complications, are at increased risk of mental health difficulties as well as being less likely to attend perinatal appointments [28–30].

One way in which perinatal services responded to the COVID-19 pandemic was by increasing the use of technology to provide existing services. Though PHCPs highlighted some benefits from the increasing use of technology, many PHCPs reflected on the obstacles both they and young parents faced utilising telemedicine. In line with findings from a survey-based study collating over 1000 responses from maternal and newborn health professionals who used telemedicine as part of their care [31], PHCPs in our study felt that aspects of their service could not be replicated online and felt there was confusion about clear guidelines for its use in terms of confidentiality and clinical governance. PHCPs also highlighted concerns that remote assessments could not provide the same level of detail as face-to-face assessments, which may lead to misdiagnosis. Though more data specifically on patient safety related to the use of telemedicine in perinatal care is needed, concerns over patient safety in relation to telemedicine have already been raised in other areas of healthcare during COVID-19 [32]. In the present study, PHCPs also identified internet and mobile data charges as a significant barrier to many young parents’ ability to engage with telemedicine. It is well established that there is a lack of parity of access to telemedicine among different socioeconomic backgrounds [33] and that these barriers, specifically internet poverty and a lack of evidence-based guidelines, need to urgently be addressed to ensure young parents and their children are not left behind.

The relationship between a healthcare professional and a young parent has long been considered vital to both the mother and the baby [34]. A facilitator for all parents, but particularly for young parents, engaging in services is a sense of connectedness with the healthcare worker. This allows a trusting therapeutic relationship to develop, as well as ensuring support is tailored to the needs of the individual [35–37]. The use of telemedicine has been found to make it more difficult for healthcare professionals to develop such relationships with service users [31]. Previous research also suggests that young mothers may be less likely to ask for help for fear of being judged by healthcare professionals and a general distrust of healthcare systems [38]. This may explain why this cohort has been found to be more likely to engage in face-to-face contacts [30, 39]. The development of a trusting relationship with a healthcare professional therefore presents another barrier for young parents coming to the attention of perinatal services. The specific challenge for these services is likely to include the nature of perinatal assessments, which by necessity touch on difficult topics such as maternal mental health and child welfare. These may be difficult to discuss openly in the absence of such a relationship and via telemedicine. This may explain why the walks instigated by PHCPs in this study were perceived as highly beneficial to both parents and PHCPs as they allowed connections to form between PHCPs and young parents.

PHCPs also felt that a reduction in face-to-face interventions such as baby groups increased young parents’ social isolation and exacerbated pre-existing mental health difficulties, such as depression and anxiety, which has been seen more broadly across perinatal populations [13]. PHCPs highlighted that online groups could not provide the same opportunities for young parents to bond and interact. These connections allow young parents to share the experience of new parenthood, receive social support, reduce feelings of isolation and derive a sense of validation of their own parenting from the interacting with other parents [40, 41]. This highlights that while online groups can provide information and educational aspects, they are limited in terms of creating social connection and support, which must not be overlooked by services.

Interestingly, PHCPs also reported some positive aspects of the use of telemedicine including parents being easier to reach at times and an increase in the frequency of contacts with service users. As it has been shown that increasing numbers of parents do access information online related the perinatal period [42] this may provide an avenue to increase the sharing of information from healthcare professionals to service users. However this should be accompanied by evidence-based guidelines addressing barriers relating to digital poverty [43]. Healthcare professionals in this study felt that in the future a blended approach would be optimal. A recent review of internet videoconferencing prior to the pandemic found that the use of telemedicine appeared to work best if patients and clinicians already had a pre-existing relationship [44], which mirrors our findings regarding the needs of young parents and the professionals working with them.

As described across other healthcare settings PHCPs also reported on professional challenges including an increased workload, changing guidelines, limited resources, difficulties faced when caring for patients affected by COVID-19 as well as occupational exposure to the virus and the loss of colleagues [45]. It is important not to neglect these factors as they have been found to adversely affect the mental health of healthcare professionals [22, 46].

### Strengths and limitations

To our knowledge this is the first UK based study looking at the impact of the COVID-19 pandemic on PHCPs, particularly those caring for young parents in a community setting. Despite PHCPs being drawn from a wide range of professional backgrounds, the majority had a background in health visiting or family nursing, meaning the experiences presented here may not fully represent all professionals providing perinatal care to young parents. The timing of the participant interview schedule in relation to the UK national lockdowns also meant it was not possible to elucidate longitudinal changes to perinatal services over time. The results presented here are reflections across services more broadly during the COVID-19 pandemic and may be affected by recall bias. Although the research team asked specifically about PHCPs experiences of working with young parents, some highlighted that they were working with parents of all ages and that it was sometimes difficult to report specifically on their experiences with young parents alone. A small number of participants were from the same clinical team; although they had individual case-loads there is a possibility of similar shared experiences during the pandemic. All PHCPs interviewed were female and further research would need to address this gender-imbalance. Most PHCPs also reflected predominantly on the experience of mothers, and it remains unclear how PHCPs felt fathers were affected by the pandemic, despite evidence showing that fathers were also adversely impacted during this time [47, 48]. Finally, this study was based in the UK and it remains unclear how these results can be generalised more broadly.

## Conclusion

This study highlights the impact that the COVID-19 pandemic had on the provision of perinatal services to young parents, who were considered by PHCPs to be particularly vulnerable to service disruptions due to pre-existing challenges faced by this cohort. Perinatal services aim to not only address the health needs of the child and the mother but also provide young parents with an avenue of social support which can help reduce social isolation. While some positives of telemedicine for service provision were identified, it was not a perfect substitute for in-person interactions. PHCPs consistently reported that young parents found accessing online support more difficult and responded best when able to establish a consistent and meaningful relationship with a healthcare professional. Crucially, this was considered more difficult to establish using telemedicine.

Findings from this paper can inform future service provision, particularly if further restrictions are necessary in light of the COVID-19 pandemic. This would include ensuring equal access to telemedicine services for all service users, providing information to new parents if they struggle with access, and the use of a blended approach of technology and face-to-face interactions moving forwards. This would allow regular contact between professionals and young parents while maintaining access to opportunities for social interactions with other parents thereby ensuring the wellbeing of the whole family.

## Data Availability

The datasets generated and/or analysed during the current study are not publicly available to protect participants, coding frames and analysis steps are available from the corresponding author on reasonable request.
